# Impact of the COVID-19 Italian Lockdown on the Physiological and Psychological Well-Being of Children with Fragile X Syndrome and Their Families

**DOI:** 10.3390/ijerph18115752

**Published:** 2021-05-27

**Authors:** Elisa Di Giorgio, Roberta Polli, Marco Lunghi, Alessandra Murgia

**Affiliations:** 1 Department of Developmental Psychology and Socialization, University of Padova, Via Venezia 8, 35131 Padova, Italy; marco.lunghi@unipd.it; 2 Molecular Genetics of Neurodevelopment, Department of Woman and Child Health, University of Padova, Via Giustiniani 3, 35128 Padova, Italy; roberta.polli@unipd.it (R.P.); alessandra.murgia@unipd.it (A.M.); 3 Fondazione Istituto di Ricerca Pediatrica (IRP), Città Della Speranza, Corso Stati Uniti 4/F, 35127 Padova, Italy

**Keywords:** COVID-19, children with fragile-X syndrome, lockdown effects, sleep problems, psychological well-being, parental self-efficacy

## Abstract

On 10 March 2020, in Italy, a total lockdown was put in place to limit viral transmission of COVID-19 infection as much as possible. Research on the psychological impact of the COVID-19 pandemic highlighted detrimental effects in children and their parents. However, little is known about such effects in children with neurodevelopment disorders and their caregivers. The present study investigated how the lockdown has impacted the physiological and psychological well-being of children with Fragile X-Syndrome (FXS), aged from 2 to 16 years, and their mothers. In an online survey, 48 mothers of FXS children reported their perception of self-efficacy as caregivers and, at the same time, their children’s sleep habits, behavioral and emotional difficulties during, and retrospectively, before the lockdown. Results showed a general worsening of sleep quality, and increasing behavioral problems. Although mothers reported a reduction in external support, their perception of self-efficacy as caregivers did not change during the home confinement compared to the period before. Overall, the present study suggested that specific interventions to manage sleep problems, as well as specific therapeutic and social support for increasing children and mother psychological well-being, need to be in place to mitigate the long-term effects of a lockdown.

## 1. Introduction

On March 11, COVID-19 was declared a pandemic by the World Health Organization (WHO) [1]. Huge efforts have been made around the world to stop the infection in order to safeguard people’s physical health. Indeed, the total lockdown imposed by the Italian Government included home confinement, movement restriction, smart working modality at home, and temporary closure of non-essential businesses and schools.

Although necessary, prolonged home confinement and social distancing might be detrimental for mental health, affecting people’s lives by disrupting their everyday behavior and daily routines. Most people are experiencing unprecedented stressful conditions that may not only increase psychological problems such as depression, stress and anxiety [2], but also impact their sleep quality, in terms of timing and duration [3,4].

Typically, developing children are not indifferent to the dramatic impact of the COVID-19 epidemic. The literature has highlighted that the pandemic outbreak, imposing a prolonged and unexpected interruption of school routine, daily activities and connections with peers, could represent an important risk factor for physical and mental health in children and their families [5,6,7,8,9]. For instance, it has been shown that prolonged home confinement and social distancing notably impacted sleep quality, time perception and behavioral well-being in pre-schoolers and school-age children and their mothers [5,7].

This sudden change in everyday behavior may be particularly challenging for families and children with special needs [10,11,12], such as in the case of Fragile X Syndrome (FXS), given the dependence of many on carefully established routines and relationships as well as professional and informal support.

FXS, an X-linked dominant disorder, is the most common inherited cause of intellectual disabilities. In the majority of cases, this genetic disorder [13] results from alterations of the FMR1 gene and the absence of Fragile X Mental Retardation Protein (FMRP) expression, leading to a characteristic phenotype including cognitive, behavioral and emotional functioning. That is, individuals with FXS have intellectual disabilities, attention-deficit/hyperactivity disorder, executive dysfunction, autistic-like features and other socioemotional problems, such as social anxiety and mood disorders [14,15].

Indeed, the response to COVID-19 could affect children with FXS who require strict adherence to routines and experience distress and anxiety when that is not possible [16], more severely than it affects most children with neurotypical development [7,9]. School closure, along with necessary measures such as self-isolation and social distancing, may be particular detrimental for children with FXS, for whom such closures mean a lack of access to the resources they usually have through schools. When schools are closed, their symptoms could relapse. Even more important, the forced school closures and the restrictions for rehabilitation centers led children with neurodevelopmental disorders to lose external and personalized professional support (e.g., delivered by motor and speech therapists, educators, developmental neuropsychiatrists) that they constantly need. Such a highly demanding and stressful situation could have a detrimental impact on children with FXS and on their parents and their sense of self-efficacy, broadly defined as the expectation caregivers hold about their ability to parent successfully [17].

Parenting self-efficacy can be considered a significant predictor of maternal perceptions of child adjustment [18], as well as a construct able to mediate the impact of children’s health on maternal well-being [19]. Indeed, behavioral and emotional problems not only have an impact on children’s life and well-being, but are also a burden for the whole family [20].

Providing parents with useful parenting tools, as well as supporting them in handling these problems, is of paramount importance, especially during the COVID-19 pandemic. However, the literature on the effects of the lockdown in children with special needs and their families is scarce and consideration has not been made for both physiological (e.g., sleep quality) and psychological problems [10]. In particular, a better understanding of how the lockdown, and especially the imposed home confinement, impacts FXS children’s physiological and psychological well-being, as well as that of their caregivers, is urgent.

To fill this gap, the present research aims at characterizing (i) the changes in FXS children’ sleep quality, emotional and behavioral problems during the lockdown compared to the period before, and (ii) the caregivers’ self-efficacy in managing such problems.

In line with previous studies [5,7,9], we hypothesize a general worsening of sleep quality, as well as increasing self-regulation difficulties and behavioral problems in children during the lockdown. Further, we expect that mothers, with the schools closed and in the absence of external support, will report greater difficulties in managing their children, with a consequence of less parental self-efficacy perception.

## 2. Materials and Methods

### Participants

Italian mothers and their children affected by FXS were involved in an online survey shared via social media for a specific time window, from April 16 to May 4, 2020. Participants were recruited through online advertisements on research-related websites and social media groups of the “*Associazione Italiana Sindrome X-Fragile Onlus*”. Inclusion criteria were: (a) being at least 18 years old, and (b) having children between 2 to 16 years of age with FXS. A total of 48 mothers (M*_age_* = 44.80 years, SD*_age_* = 6.78, age-range = 30–51 years) fulfilled the survey. The mean age of children (n = 53, of which 45 males) was 9.71 years (SD*_age_* = 4.15, age-range 3–16) (see Table 1 for descriptive data). Five out of 48 mothers involved in the present research had more than one child with FXS, therefore they fulfilled the survey for both children.

## 3. Procedure

After the participants had provided informed consent (the project was approved by the Ethical Committee of Psychological Research of the University of Padova (Prot. No. 3573), they responded to the first part of the survey, which comprised some general questions about socio-demographic characteristics such as gender, age, education, and employment, as well as some questions related to COVID-19 (e.g., where they obtain information about the virus or how frequently they search for COVID-19 information). In the second part of the survey, mothers were asked to describe their children’s sleep quality, daily routines, and behavioral and emotional problems, as well as their own feelings about their self-efficacy as caregivers. They had to respond to the questions relative to the present, that is the period during the lockdown (from 16 April to 4 May, after five weeks of confinement), and retrospectively to the week before (i.e., last week of February from 24 to 29 of February). The total time to complete the survey was about 25 min. The project will also include a follow-up where the same survey will be presented again at the end of the emergency.

## 4. Measures

### 4.1. Sleep Quality, Daily Routines and External Support Perception Information

Caregivers were asked ad-hoc created questions about their children’s sleep habits (e.g., “*How frequent are night awakenings*?”) and daily routines (e.g., “*Is your child involved in speech therapy?*” or “*Is your child involved in occupational therapy*?”). Further, caregivers’ own perception about the support received in the care and management of their child was addressed (e.g., “*How much support do you feel in the management of your child by teachers?*”).

Non-parametric tests (e.g., chi-square) were used to compare the frequency of responses between the two time-points, before and during the lockdown (see Supplementary Files for a detailed description of the items).

### 4.2. Child Adjustment and Parent Efficacy Scale-Developmental Disability (CAPES-DD)

The Italian version of the Child Adjustment and Parent Efficacy Scale-Developmental Disability (CAPES-DD) [21,22] was employed to assess behavioral (Behavioral Problems subscale, 10 items) and emotional (Emotional Behavior Subscale, 3 items) problems in children with developmental disabilities aged 2- to 16-years, as well as caregivers’ self-efficacy in handling these problems (Self-Efficacy Scale, 16 items). A Total Problem Scales was derived by the sum of the Behavioral Problems subscale, the Emotional Behavior Subscale plus three Additional Items (e.g., “Fusses or refuses to eat”). In addition, this brief inventory also assessed the children’s prosocial behaviour (Prosocial Behaviour Scale, 8 items). Examples of items are “my child loses their temper, or “hurts me or others (e.g., hits, bites, scratches, pinches, pushes)” or my child “seems unhappy or sad” or “cooperates with self-care routines”. With its very economical length of 24 items, the CAPES-DD has the advantage of being completed briefly by parents. For the Intensity and Prosocial Scales, the caregiver rated each item from 0 (“Not true of my child at all”) to 3 (“True of my child most of the time”). As an example of items for the Self-Efficacy scale, parents’ confidence is rated for the difficulties from 1 (“Certain I can’t manage it”) to 10 (“Certain I can manage it”).

## 5. Results

Demographic characteristics for the 48 mothers who completed the survey and their children with FXS are shown in Table 1.

### 5.1. Sleep Quality, Daily Routines and External Support Perception Information

As expected, the sleep pattern was markedly affected by the lockdown. Specifically, mothers reported a worsening with regard to falling asleep difficulties (*χ^2^* = 21.56, *p* < 0.001), with an increase in the falling asleep time (*χ^2^* = 86.01, *p* < 0.001) and in the frequency of night awakenings (*χ^2^* = 41.29, *p* < 0.001).

Children’s daily routines changed, with an important reduction of some of the most relevant healthcare services, such as speech, psychomotor and occupational therapy. Sport, educational and peer activities (e.g., scouts) were also reported as markedly reduced during the lockdown (all differences were statistically significant, with *p* < 0.001, see Table 1).

Finally, the measures put in place to contain the epidemic have drastically reduced the caregivers’ possibility of getting help from the outside (grandparents, friends, teachers, etc. (see Supplementary Materials)). That is, mothers’ perception about the support received in the care and management of their children markedly changed during lockdown (Table 2).

### 5.2. CAPES-DD

**Figure 1:**
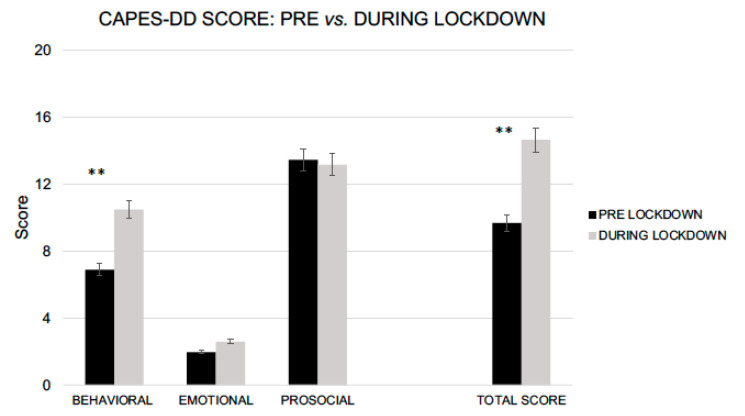
CAPES-DD scores between pre and during lockdown (** *p* > 0.01).

Focusing on the CAPES-DD, children showed an increased in the Total Problem Scale (*t_52_* = 3.19, *p* = 0.003, Cohen’s *d* = 0.46; pre-lockdown, *M_score_* = 9.69, *SD_score_* = 6.77 vs. during lockdown, *M_score_* = 14.65, *SD_score_* = 10.97). Mothers reported more behavioral problems in their children during the lockdown compared with the period immediately before restrictions (Behavioral Problems Subscale, *t_52_* = 3.33, *p* = 0.002, *d* = 0.48; pre-lockdown, *M_score_* = 6.90, *SD_score_* = 5.08 vs. during lockdown, *M_score_* = 10.49, *SD_score_* = 7.63). No differences were found for either the Emotional Behavior Subscale, with *t_52_* = 3.19, *p* = 0.066 (pre-lockdown, *M_score_* = 1.96, *SD_score_* = 1.44 vs. during lockdown, *M_score_* = 2.59, *SD_score_* = 2.10), or the Prosocial Behavior Scale (*t_52_* = 0.548, *p* = 0.586 pre-lockdown, *M_score_* = 13.45, *SD_score_* = 5.31 vs. during lockdown, *M_score_* = 13.16, *SD_score_* = 5.36) (Figure 1).

Although mothers reported higher children’s behavioral problems, their self-efficacy in managing these problems remained stable between pre- (Self-Efficacy Scale, pre-lockdown, M *M_score_* = 104.39, *SD_score_* = 45.06) and during lockdown (*M_score_* = 105.59, *SD_score_* = 41.66), *t_52_* = 0.264, *p* = 0.793.

## 6. Discussion

The aim of the present study was to evaluate the impact of five weeks home confinement during the Italian lockdown in children and adolescents with FXS in terms of sleep habits and behavioural well-being, as well as in their mothers’ self-efficiency perception in managing the emerged difficulties.

In line with our initial hypotheses, data showed that restrictive measures had negative effects on children’s daily routines, which in turn was contributing to alter sleep habits. Specifically, we observed a worsening in regard to falling asleep difficulties, with an increase in the frequency of night awakenings and falling asleep. Although in line with previous studies on the worsening of sleep quality in children during the lockdown [5,7], this aspect is particularly detrimental for children and adolescents with FXS, for whom sleep disturbances are often a characteristic condition of FXS syndrome [23].

In regard to psychological well-being, concerning the period before the lockdown, mothers reported a general worsening of some difficulties in their children. They perceived them as more hyperactive, with a need for a greater demand for attention, as well as with a worsening inhibitory self-control capacity, which in turn led to increased frequency of oppositive behaviors. On the other hand, no differences emerged with regard to emotional and social aspects.

A breakdown in children daily routines, as well as their experience of a drastic reduction in healthcare services, can be considered as a main factor for their physiological (e.g., sleep quality) and psychological outcomes (e.g., behavioral problems). This interpretation is in line with the idea that a structured and pre-planned day [24] is one of the most protective factors for psychological well-being. Therefore, for FXS children and their families having a scheduled routine is crucial to limit problem behavior [16,25].

During the national lockdown, without external support usually delivered by educators, psychologists and therapists, parents had to reorganize and structure daily routines in accordance with their children’s needs.

Interestingly, contrary to our initial hypothesis, although mothers reported a substantial reduction of the external support together with a general worsening of some difficulties in their children, they did not perceive themselves as less effective in managing such difficulties. Indeed, the parental self-efficacy perception remained stable during the home confinement compared to the period immediately before the lockdown.

This is a very interesting result in light of the literature that defines the parental self-efficacy perception as a protective factor, even for parents with children with special needs. Broadly speaking, parents with high self-efficacy experience less anxiety and stress, are more optimistic, effective in problem-solving and collaborative with services [26]. Even more important, parental self-efficacy perception can be enhanced through intervention, translating into improved child, parent and service outcomes [27].

Nevertheless, such a result may be due to a sampling bias, since participants were recruited through associations of parents with FXS children and FXS associations. This means that experiences of families who are connected to territorial services and parent associations may differ from those who are not affiliated with them. Therefore, parents who are already part of an association could be more sensitive and informed about the factors that promote psychological well-being, such as the importance of the perception of parental self-efficacy. Future studies should also involve those families with children and adolescents with FXS who are not affiliated with such associations.

The aim of the present research was to provide a real-time picture of the situation for families with special needs children in Italy. Although the topic is relevant, some limitations have to be considered. First, the small sample of the present study cannot be considered representative of all the mothers of children with FXS in the Italian population. Second, the present research focused only on mothers, as they are in most cases the primary caregivers for children. Therefore, future studies should also consider involving the other parent, who is directly involved in raising the children. Third, children’s behavioral and emotional difficulties were not directly measured but reported by their mothers. Fourth, future studies on the COVID-19 effect on FXS children should use standardized and accepted questionnaires for sleep quality. Finally, as did previous studies on the COVID-19 pandemic [4,28], we used retrospective questions to compare the situation during the lockdown to a baseline before the outbreak. This method is not without limitations and bias, however some previous studies suggested that a retrospective method is quite consistent [29].

## 7. Conclusions

COVID-19 is a challenge to the daily routine of children, especially those with neurodevelopmental disabilities such as FXS. The results suggested that the lockdown is particularly challenging for mothers and their children with special needs, given the reliance on carefully established daily-routines, relationships, healthcare services and external support. Effective measures need to be in place to mitigate the lockdown effects. In regard to sleep problems, the task force of the European CBT-I Academy [3] suggested several guidelines for managing such problems to reduce stress and possibly prevent behavioral and emotional difficulties in children and in their mothers. In line with the structured day hypothesis [24], it is important to structure daily life activities. For increasing psychological well-being, prevention and intervention programs, together with specific therapeutic and social support, should be implemented. Finally, parents would benefit from professionally supported interventions targeting parenting competence, in order to maintain and/or improve their self-efficacy as caregivers. Such interventions would positively affect the adaptive capacities of children and reduce child-related parenting stress.

## Figures and Tables

**Table 1:** Demographics, children’s sleep quality and daily routines.

MOTHERS		N (%)
Diagnosis	Full Mutation	8 (17)
	Premutation	30 (62)
	Mosaicism	10 (21)
Work condition	Smart working	18 (38)
	Stopped	8 (16)
	No work	13 (27)
	Regular	9 (19)
COVID-19 information (How often…?)	Never	2 (4)
	Sometimes	31 (65)
	Often	13 (27)
	Frequently	2 (4)
COVID-19 information (Where…?)	TV	45 (94)
	Daily Newspapers	31 (65)
	Internet	24 (50)
	Social Media	19 (40)
**CHILDREN**		**N (%)**
Diagnosis	Full Mutation	42 (79)
	Premutation	2 (4)
	Mosaicism	9 (17)
Covid-19 information (How…?)	Fairy Tails	15 (28)
	Video	20 (38)
	Scientific Information	13 (25)
	Family	45 (85)
**CHILDREN SLEEP QUALITY**		
	BEFORE LOCKDOWN N (%)	DURING LOCKDOWN N (%)
Difficulty falling asleep	Never 31 (58)	24 (45)
	Sometimes 19 (36)	21 (40)
	Often 3 (6)	8 (15)
Time to fall asleep	>60 min 1 (2)	4 (7)
	45–60 min 4 (7)	7 (13)
	30–45 min 7 (13)	10 (19)
	15–30 min 21 (40)	18 (34)
	<15 min 20 (38)	14 (27)
Hours sleeping per night	8–11 h 39 (73)	39 (73)
	5–8 h 13 (25)	13 (25)
	<5 h 1 (2)	1 (2)
Number of times of night awakenings	Never 27 (51)	24 (45)
	<2 times 21 (40)	21 (40)
	2–4 times 5 (9)	6 (11)
	>4 times 0 (0)	2 (4)
Difficulty waking up in the morning	Yes 10 (19)	8 (15)
	No 43 (81)	45 (85)
**CHILDREN DAILY ROUTINES**		
	BEFORE LOCKDOWN N (%)	DURING LOCKDOWN N (%)
Speech Therapy	21 (40)	7 (13)
Psychomotor Therapy	27 (51)	10 (19)
Occupational Therapy	11 (21)	3 (6)
Sport Activities	35 (66)	6 (11)
Educational and peer Activities	20 (38)	9 (17)

**Table 2:** Non-parametric statistics on the caregivers’ perception about the external support received in their children’s management.

	*χ^2^*	*p*
Family (grandparents)	40.82	<0.001
Friends	18.02	0.001
X-Fragile Associations	49.25	<0.001
Educators	35.76	<0.001
Teachers	30.15	<0.001
Other (Doctors)	31.32	<0.001

## Data Availability

The data presented in this study are available on request to the authors.

## Supplementary Materials

The following are available online at https://www.mdpi.com/article/10.3390/ijerph18115752/s1.
