# Supplementary material for: Impact of the COVID-19 Italian Lockdown on the Physiological and Psychological Well-Being of Children with Fragile X Syndrome and Their Families
Source: Int J Environ Res Public Health. 2021 May 27;18(11):5752. doi: 10.3390/ijerph18115752 (PMC8199386; doi:10.3390/ijerph18115752)
Supplement: Supplementary file 1 [file ijerph-18-05752-s001.zip › ijerph-1203065-supplementary.pdf]

International Journal of
Environmental Research
and Public Health

MDPI

Supplementary Files

"Impact of the COVID-19 Italian lockdown on the physiological and psychological well-being of children with Fragile X Syndrome and their families"

Elisa Di Giorgio, Roberta Polli, Marco Lunghi & Alessandra Murgia

AD-HOC QUESTIONNAIRE

COVID INFORMATION SECTION

How often do you look for information about the COVID-19 infection during the day?	Never	Sometimes	Often	Frequently
In which of the following media are you looking for information about the COVID-19?	TV	Daily Newspaper	Internet	Social Media

*SLEEP QUALITY SECTION**

How often did your child have difficulty falling asleep?	Never	Sometimes	Often		
How long does it take your child to fall asleep?	More than 60 minutes	Between 45 and 60 minutes	Between 30 and 45 minutes	Between 15 and 30 minutes	Less than 15 minutes
On average, how many hours does your child sleep per night?	8-11 hours	5-8 hours	Less than 5 hours		
How frequent are night awakenings?	Never	Less than 2 times	Between 2 and 4 times	More than 4 times	

Does your child have difficulty waking up in the morning?	Yes	No			

*EXTERNAL SUPPORT PERCEPTION SECTION**

	Little	Enough	A lot
How much support do you feel in the management of your child by **other relatives (e.g., grandparents)**?	1	2	3
How much support do you feel in the management of your child by **your friends**?	1	2	3
How much support do you feel in the management of your child by **X-Fragile Association**?	1	2	3
How much support do you feel in the management of your child by e**ducators**?	1	2	3
How much support do you feel in the management of your child by **teachers**?	1	2	3
How much support do you feel in the management of your child by **other significant figures**?	1	2	3

*CHILDREN DAILY ROUTINES SECTION **

Is your child involved in speech therapy?	Yes	No
Is your child involved in psychomotor therapy?	Yes	No
Is your child involved in occupational therapy?	Yes	No
Is your child involved in sport activities?	Yes	No
Is your child involved in educational and peer activities (i.e., scouts)?	Yes	No

* Caregivers have to respond to these questions two times, thinking to the present during the lockdown, and retrospectively to the period before the lockdown.
